# Detection of *Mycobacterium tuberculosis* complex infection in Asian elephants (*Elephas maximus*) using an interferon gamma release assay in a captive elephant herd

**DOI:** 10.1038/s41598-020-71099-3

**Published:** 2020-09-03

**Authors:** Songkiat Songthammanuphap, Songchan Puthong, Chitsuda Pongma, Anumart Buakeaw, Therdsak Prammananan, Saradee Warit, Wanlaya Tipkantha, Erngsiri Kaewkhunjob, Wandee Yindeeyoungyeon, Tanapat Palaga

**Affiliations:** 1 Graduate Program in Microbiology and Microbial Technology, Department of Microbiology, Faculty of Science, Chulalongkorn University, Phayathai Road, Pathumwan, Bangkok, 10330 Thailand; 2 Center of Excellence in Immunology and Immune-Mediated Diseases, Chulalongkorn University, Phayathai Road, Pathumwan, Bangkok, 10330 Thailand; 3 Institute of Biotechnology and Genetic Engineering, Chulalongkorn University, Phayathai Road, Pathumwan, Bangkok, 10330 Thailand; 4 Inter-Department Graduate Program in Biotechnology, Faculty of Science, Chulalongkorn University, Phayathai Road, Pathumwan, Bangkok, 10330 Thailand; 5 The National Center for Genetic Engineering and Biotechnology, National Science and Technology Development Agency (NSTDA), 113 Thailand Science Park, Phahonyothin Road, Khlong Nueng, Khlong Luang, Pathum Thani, 12120 Thailand; 6 The Zoological Park Organization of Thailand, Bureau of Conservation and Research, Pracharat Sai 1 Road, BangSue, Bangkok, 10800 Thailand

**Keywords:** Microbiology, Zoology, Diseases

## Abstract

Tuberculosis is highly contagious disease that can be transmitted between humans and animals. Asian elephants (*Elephas maximus*) in captivity live in close contact with humans in many Asian countries. In this study, we developed an interferon gamma release assay (IGRA) for elephant TB detection using antigens from the MTB complex (MTBC) and nontuberculous mycobacteria (NTM) as stimulating antigens (PPD, ESAT6, CFP10) to elicit a cell-mediated immune response (CMIR). The developed assay was applied to an elephant herd of more than 60 animals in Thailand, and the results were compared with those obtained through serological detection. IGRA has sufficient sensitivity for detecting elephant interferon gamma (eIFNγ) from specific antigen-stimulated PBMCs. Among 60 animals tested, 20 samples (33.3%) showed negative results for both MTBC and NTM infection. Eighteen samples (30%) showed positive responses against PPD from *M. bovis* and/or ESAT6 and CFP10, indicating MTBC infection. In contrast, only 15.6% showed seropositivity in a commercial serological test kit for elephant TB. The discrepancies between serological and CMIR highlight that the two methods may detect different stages of elephant TB. Therefore, employing both tests may enable them to complement each other in correctly identifying elephants that have been exposed to MTBC.

## Introduction

*Mycobacterium tuberculosis* (M. tb) is reported to be transmitted from animals to humans or vice versa. Except for humans, who are known as natural reservoir hosts for M. tb, various kinds of animals, such as elephants, nonhuman primates, psittacine birds, cattle, and tapirs, have been reported to be infected with M. tb by interacting with humans^1–4^. The infection can cause tuberculosis (TB) in these animals. Moreover, the infected animals will become the source of the pathogens and have the potential to transmit this disease back to humans or to other animals. The main cause of TB infection in animals can come from prolonged contact with humans with active TB, especially in captive elephants, which show high susceptibility to M. tb infection^5^. The susceptibility to M. tb infection of the elephants depends on their species. According to the report by Une and Mori in 2007, Asian elephants (*Elephas maximus*) are much more susceptible to M. tb infection than are African elephants (*Loxodonta africana*); therefore, they are at high risk of becoming infected and may spread it to humans^6^.

TB in elephants is often a chronic infection and often does not show clinical symptoms until it reaches an active stage of disease^7^. Infected elephants might show fatigue symptoms, do not work properly, may have mucus, exhibit labored breathing and weight loss, and eventually die^8^. Hence, an accurate diagnostic approach covering all phases of M. tb infection is required for preventing the epidemic of TB in elephants and the transmission between the infected animal and human and among the elephants. Because elephants are large in size and have thick skin, the common M. tb diagnoses that are used in humans, such as chest X-ray and tuberculin skin tests, are not practical. Moreover, bacterial culture from trunk washes, which is the gold standard for elephant TB diagnosis, has low sensitivity, is time-consuming and can detect TB only in the active stage^9^. Diagnostic assays detecting humoral immune response (HIR) as antibodies specific for MTB complex antigens in serum have limitations in yielding false positive results from cross-reactivity with environmental mycobacteria^10^.

The Mycobacterium genus can be divided into two groups, i.e., *Mycobacterium tuberculosis* complex (MTBC) and nontuberculous mycobacteria (NTM). MTBC is a group of Mycobacterium species that can cause tuberculosis in humans and animals^11^. NTM is a group of the genus Mycobacterium that does not cause tuberculosis or leprosy and known as environmental mycobacteria that can be commonly found in soil and water. MTBC and NTM can be diagnosed and differentiated by using MTBC-specific proteins as antigens in the recall response by immunological assays.

As M. tb is an intracellular pathogen, infection triggers a cell-mediated immune response (CMIR). Interferon gamma (IFNγ) is a cytokine that is produced mostly by T helper 1, cytotoxic T lymphocytes and natural killer cells and plays important roles in controlling mycobacterial infection. For this reason, the interferon gamma release assay (IGRA) in response to M. tb antigen stimulation is an alternative approach for tuberculosis diagnosis. IGRA is an in vitro blood-based test of CMIR against M. tb-specific antigens. At present, in addition to being used in humans, IGRA has been developed for tuberculosis diagnosis in many species, such as cattle, lions and deer^12,13^.

Previous studies show that IGRA can be used as a tuberculosis diagnosis tool for Asian and African elephants^14,15^. However, IGRA for elephant TB is not commercially available, and the evaluation for the use of IGRA in larger captive elephants has not been reported. In this study, we developed IGRA to detect M. tb infection that can distinguish NTM and MTBC infection in elephants. Moreover, the developed test was applied in the samples of more than 60 elephants, which is the largest to date in total numbers of elephants subjected to IGRA.

## Results

### Development of IGRA for eIFNγ

**Figure 1:**
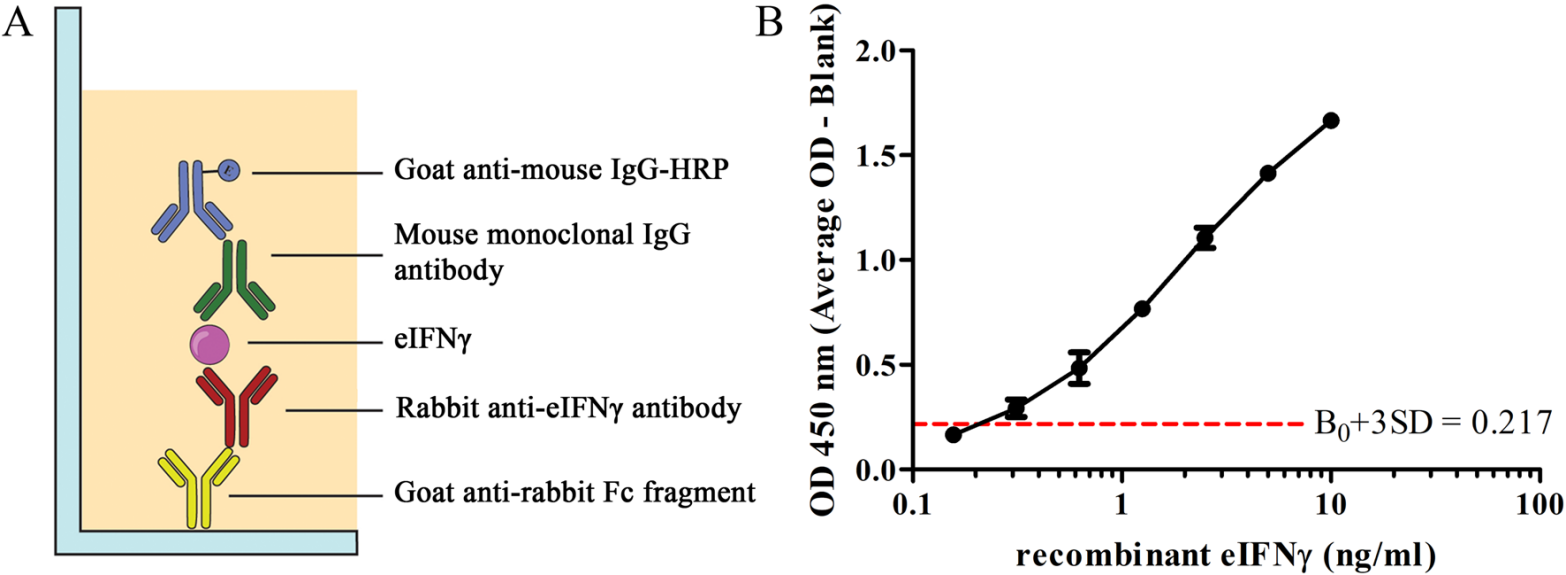
Development of sandwich ELISA to detect eIFNγ. (**A**) Schematic representation of the developed sandwich ELISA. (**B**) Standard curve of recombinant eIFNγ using rabbit polyclonal capture antibody and mouse detection antibody. The limit of detection of sandwich ELISA was estimated at 0.241 ng/mL.

To detect and quantify the level of IFNγ produced by specific antigen-stimulated T cells in peripheral blood mononuclear cells (PBMCs), we optimized and developed a sandwich ELISA using eIFNγ-specific rabbit polyclonal antibody and eIFNγ-specific mouse monoclonal antibody as capture and detection antibodies, respectively (Fig. 1A). Generation of antibody and optimization of the sandwich ELISA are detailed in “Methods” (Supplementary Figs. 1, 2). As shown in Fig. 1B, the developed sandwich ELISA exhibited a detection range between 0.3 and 10 ng/mL with a minimal OD value of B0 + 3SD at 0.217. Therefore, the limit of detection (LOD) of this ELISA is estimated at 0.241 ng/mL. Furthermore, the precision of the assay in terms of the percentage of coefficient of variation (%CV) between inter- and intra-assay were in the ranges of 5.65–12.31 and 0.78–2.50, respectively. Taken together, these results indicate that the sandwich ELISA developed in this study exhibits reproducibility and acceptable variation.

**Figure 2:**
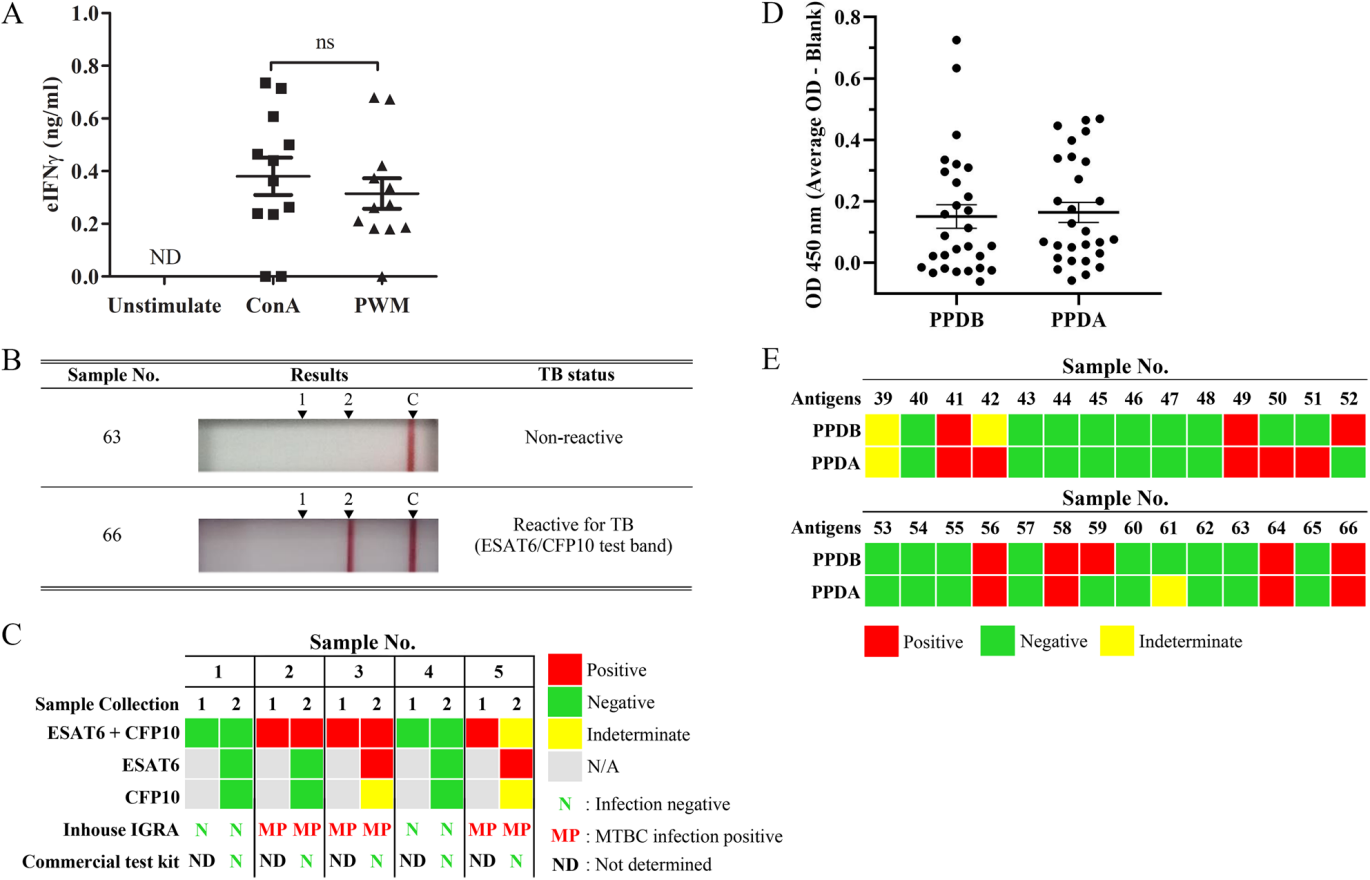
Evaluation of IGRA for MTBC detection. (**A**) The amount of eIFNγ produced from PBMCs after stimulation with mitogen for 72 h (n = 12). *ns* not significant with *p*-value > 0.05, *ND* not detected. (**B**) Serological detection by DPP VetTB assays. The images represent the results obtained for nonreactive and reactive samples from confirmed cases of TB in elephants. (**C**) The follow-up test using IGRA and DPP VetTB assay. (**D**) The response of PBMCs against PPDB and PPDA by using IGRA.

We next aimed to establish a positive control for IGRA by testing two different polyclonal mitogens against elephant PMBC. Concanavalin A (ConA) and pokeweed mitogen (PWM) were added to PBMC culture for 72 h, and the amount of eIFNγ was measured by developed sandwich ELISA. As shown in Fig. 2A, both mitogens significantly induced eIFNγ production, compared to unstimulated cells. Although the differences between ConA and PWM did not reach statistical significance, a slight increase in eIFNγ was observed in the ConA-treated group. Therefore, ConA was chosen as a positive control in the IGRA for the real sample test.

### Evaluation of IGRA using different stimulating antigens

To induce an immunological recall response against MTBC, specific antigens were tested. In this study, recombinant ESAT6 and CFP10 were included as purified specific antigens of MTBC. Blood samples were taken from five elephants with unknown MTBC infection status at two different time points (3 months apart). Recombinant ESAT6 and CFP10 were added separately or together to the elephant PBMC culture, and the amount of eIFNγ was measured by ELISA. In parallel, plasma from these samples was applied to commercial test kit DPP VetTB assays for serological detection. Representative positive test results and negative test results from the DPP VetTB assay are shown in Fig. 2B. Sample No. 66 is included as a positive control as this elephant was previously diagnosed with MTB infection using trunk wash culture^16^. Because the line on the DPP VetTB assay could be visibly detected for the serum sample No. 66, the result indicates that serological detection correctly identified infected elephant. Among the five samples tested, all showed negative results by the commercial test kit while the control sera from No. 66 showed positive result. For IGRA using ESAT6 in combination with CFP10, three samples showed consistent positive results (Fig. 2C). Among these three samples, when ESAT6 or CFP10 was used alone, two out of three samples were positive. ESAT6 stimulation resulted in a clearer positive response than CFP10 stimulation. In addition, one sample showed an indeterminate readout because the amount of eIFNγ was between the LOD and the lowest concentration of the standard curve. With the small numbers of tested samples, we detected discrepancies between the serological and IGRA tests.

Next, we tested whether PPD added as stimulating antigens in IGRA is beneficial in determining whether the animals are infected with MTBC or NTM. For this purpose, we used PPDA as a representative NTM crude antigen and PPDB as a representative MTBC crude antigen. Sample No. 66 was also included as a positive control. The OD readings of the ELISA are summarized in Fig. 2D and based on this ELISA results, the possible infection status was predicted into three outcomes, i.e. positive, negative and indeterminate. As shown in Fig. 2E, among the 27 samples tested, 15 samples (55.6%) showed negative results, while 5 samples (18.5%) yielded positive responses against both PPDA and PPDB. In contrast, 3 samples (11.1%) and 2 samples (7.4%) yielded positive results for only PPDA or PPDB, respectively. Two sample showed indeterminate readings for both antigens or PPDA alone (7.4%).

**Figure 3:**
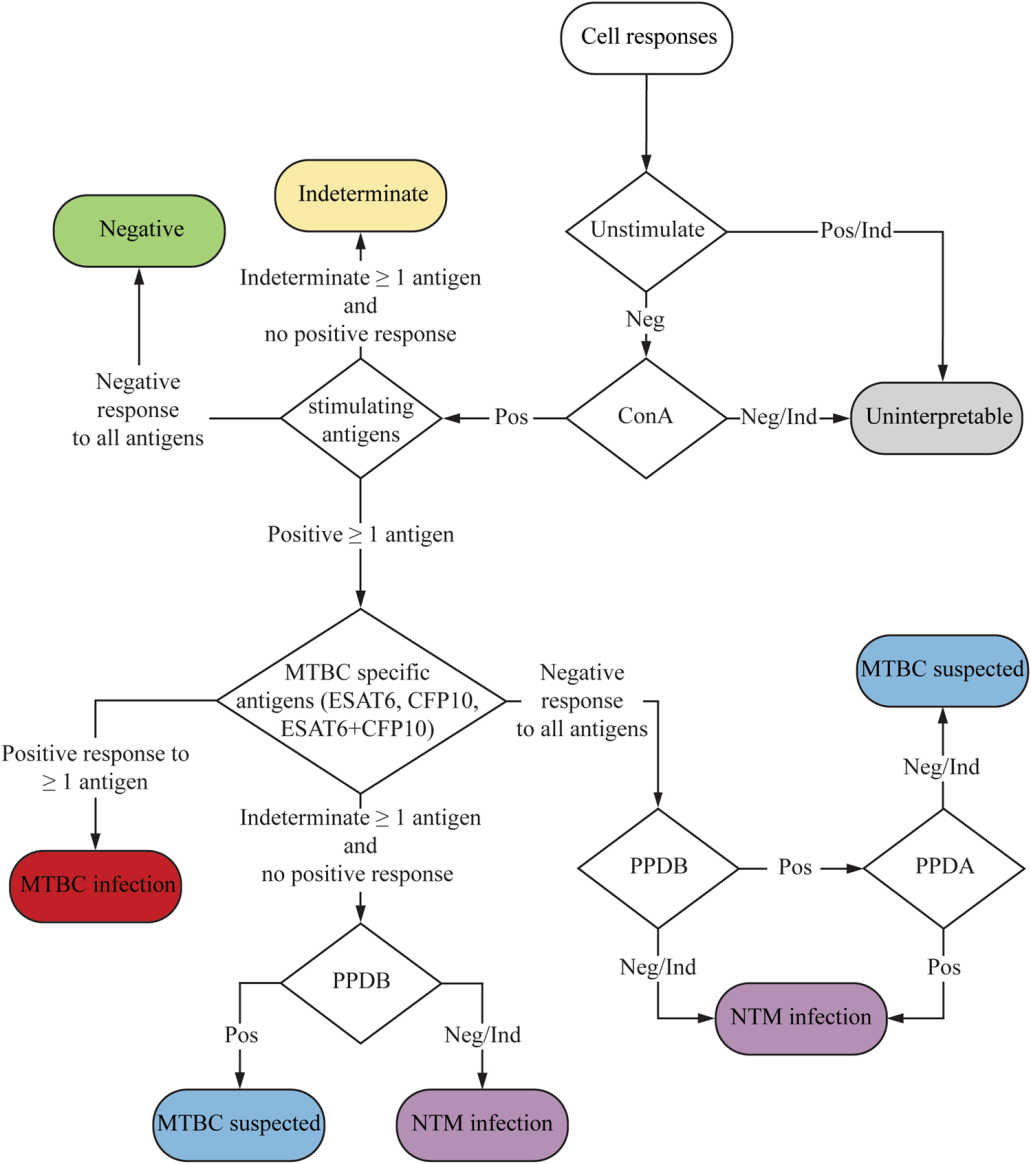
Flowchart depicting the interpretation of results from IGRA. The response of PBMC against the stimulating antigens was divided into three categories;  Negative (Neg) inducated that eIFNγ was not detectable within sample, Positive (Pos) indictaed that the amount of eIFNγ is higher than the cutoff value and Indeterminate (Ind) indicated that the amount of the eIFNγ is between the cutoff and the lowest concentration of the standard curve.

From this preliminary test result, we set up criteria for interpreting the results from IGRA, as shown in Fig. 3. IGRA using the combination of PPDA, PPDB, and MTBC specific antigens (ESAT6/CFP10) as stimulating antigens for eliciting a response, enables us to distinguish among uninfected status, MTBC infected status, NTM infected status or MTBC infected suspect status. For samples that give borderline responses [between the LOD (cutoff) and the lowest concentration points of the standard curve], they are categorized as indeterminate.

### Using IGRA in a large cohort of Asian elephants

Next, we applied the developed IGRA with the aforementioned criteria in samples from a larger cohort of Asian elephants (n = 60) in captivity. All of these elephants live in close contact with humans in rural area of Thailand or in the zoo in Thailand. The plasma of some of these samples was subjected to serological testing of the DPP VetTB assay. Among these samples, sample No. 66 (asterisk) was included in this study as a positive control^16^. As shown previously in Fig. 2, this sample showed consistent positive results for MTBC infection in the IGRA and the serological test of DPP VetTB assay.

**Figure 4:**
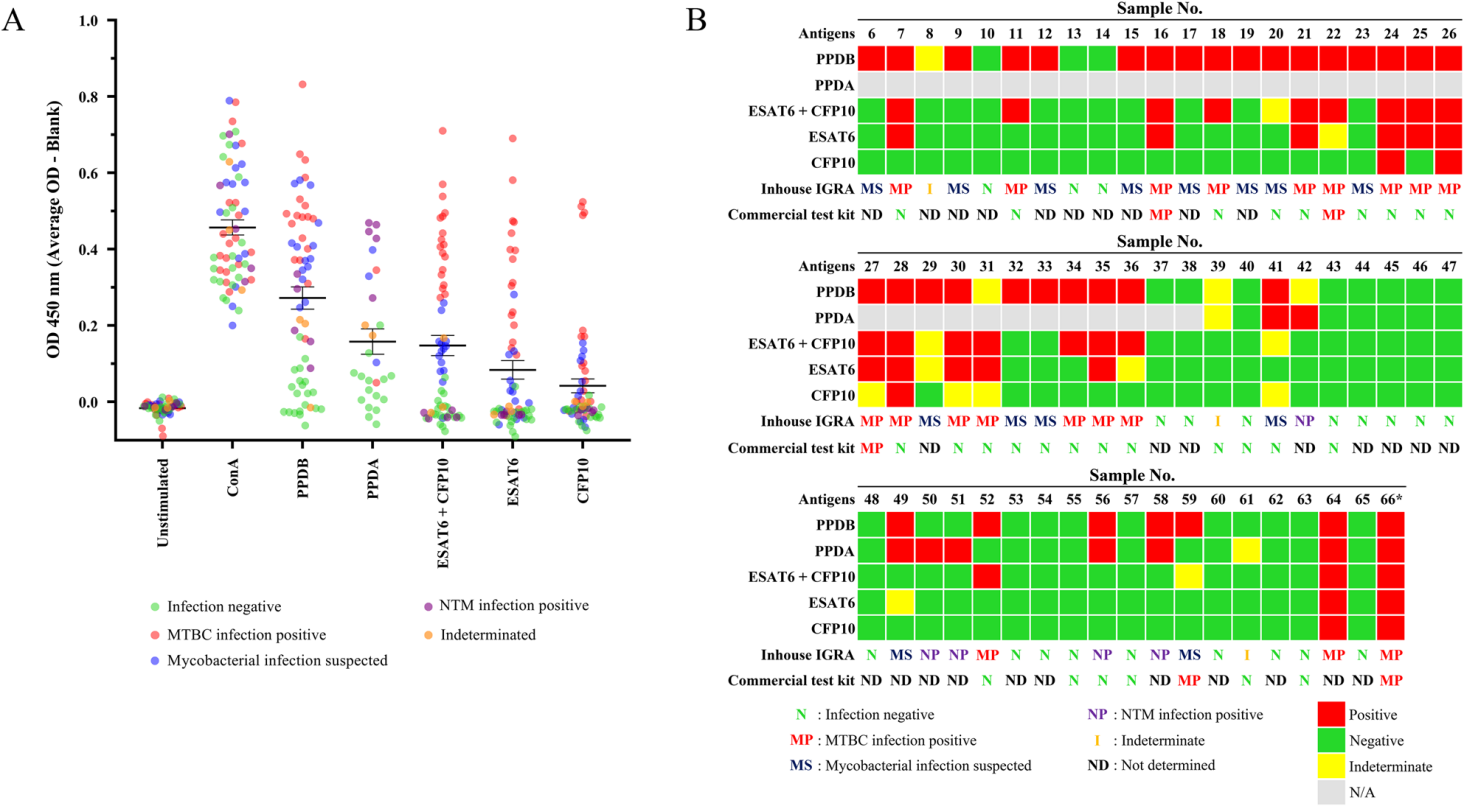
Summary of PBMC response to stimulation antigens in IGRA and DPP VetTB assays. (**A**) OD readings from ELISA of all samples are shown. Different color-coded dots represent individual elephant with the determined outcome of IGRA. (**B**) Summary of the results obtained by IGRA and serological test by DPP VetTB assay in large cohort of elephants. Asterisk indicates a positive control with positive bacterial culture result. Sixty elephant samples were tested with IGRA. Among 60 samples, 31 samples were tested with the DPP VetTB assay parallel with IGRA.

Using the criteria described in Fig. 3, the results of the IGRA are summarized in Fig. 4A,B. The ELISA OD reading results are presented in Fig. 4A while the infection status for each sample are summarized as a heat map in Fig. 4B. The calculated eIFNγ is summarized in the Supplementary Fig. 3. Among 60 samples tested, 20 samples (33.3%) showed negative results for both MTBC and NTM infection. Eighteen samples (30%) showed positive or indeterminate responses to PPDB and positive responses to ESAT6 and/or CFP10, indicating MTBC infection. Among 27 samples that were stimulated with PPDA, 5 samples (18.5%) showed positive responses to only PPDA or both PPDA and PPDB, but not ESAT6/CFP10, suggesting an NTM infection. Regardless of the responses to PPDA, 14 samples (23.3%) showed positive response to only PPDB with negative or indeterminate response against ESAT6/CFP10 thus interpreting as MTBC suspected. This result is interpreted as the animals are suspected of mycobacterium infection that may need further follow up or monitoring. Two samples responded as indeterminate for PPDB. Overall, combining the negative (20 samples out of 60 samples) and NTM (5 samples out of 60 samples) together, this cohort showed 41.7% of MTBC negative elephants (Table 1).

**Table 1:** Summary of IGRA test results.

Response (%)	Stimulating antigens				
	PPDB	PPDA	ESAT6 + CFP10	ESAT6	CFP10
Negative	38.3 (23)	63.0 (17)	63.3 (38)	73.3 (44)	86.6 (52)
Indeterminate	6.7 (4)	7.4 (2)	6.7 (4)	6.7 (4)	6.7 (4)
Positive	55.0 (33)	29.3 (8)	30.0 (18)	20.0 (12)	6.7 (4)

The numbers in the blanket are the number of the samples that responded to each antigen.

Among these samples, the ELISA results obtained when using individual ESAT6 or CFP10 or both antigens in combination, are shown in Supplementary Fig. 4. Comparing ESAT6 and CFP10 single antigen revealed that ESAT6 elicited more robust IFNγ release than CFP10 and the combination of both antigens yielded much higher level of IFNγ than when using either single antigen. This result indicated that combining the two antigens elicited stronger IFNγ responses that may increase the sensitivity of the assay.

To compare the CMIR to the serological test, 31 samples among these 60 samples were randomly subjected to DPP VetTB assay. To our surprise, only 4 out of 31 samples (15.6%) showed a positive serological response, while IGRA yielded 17 positive results (54.8%) for MTBC infection among these samples. Three out of four samples that were serological positive were also positive in IGRA. This result suggests that the serological response and the CMIR against MTBC antigens in elephants have discrepancies and may reflect different disease stages of tuberculosis in elephants.

## Discussion

In this study, we developed an IGRA for detecting MTBC and NTM infection for Asian elephants in captivity that live in close proximity to humans in Thailand. The numbers of elephants subjected to IGRA in the current study is the world largest to date. Tuberculosis is known to be transmitted from humans to animals, and elephant TB is shown to be transmitted back to humans in close contact^17^. IGRA has been used widely for TB diagnosis in humans because of its sensitivity and ability to distinguish between BCG-vaccinated and M. tb-infected individuals. For animal TB, IGRA has been experimentally performed in wild and captive animals, but none is in routine use and commercially available^18^.

The gold standard of TB diagnosis by trunk wash culture in elephants has serious limitations due to the difficulty of collecting samples and its low sensitivity. Elephants need to be trained to provide samples and the collecting procedures are accompanied with health risk to human involved^19^. More importantly, the trunk wash culture method only detects active TB where the bacteria are shed in mucus, and the negative culture results do not guarantee that the animals are free of TB. IGRA presents an alternative method for elephant tuberculosis diagnosis because the assay detects the immunological recall response to previous exposure to M. tb. IGRA for elephant TB diagnosis has been reported previously, but none was tested in large numbers of elephants, as in our study^14,15^. A study by Angkawanish et al. used ESAT6 and CFP10 together with PPDB and PPDA for stimulation. They found that the combination of stimulating antigens yielded positive response from white blood cells of elephants^14^. However, this work did not apply the test assay in larger cohort of elephants and the applicability of the assay was not determined. In this study, we aimed to investigate the potential usage of IGRA for the survey of MTBC exposed elephants, but not to diagnose active TB in elephants. Therefore, the trunk wash culture method was not performed. The sample of one elephant was included as a positive control in our study because the trunk wash from this animal has been subjected to M. tb culture and the animal has been diagnosed with TB^16^.

By using a combination of stimulating antigens, IGRA developed in this study is able to distinguish at least between NTM and MTBC infection. Our findings from IGRA indicated that 30% of elephants in this study have been previously exposed to MTBC. As MTBC infection can be latent or active, it cannot be concluded whether IGRA-positive elephants in this study actively shed bacteria. In this study, antigens in the form of PPD (crude antigens) and recombinant proteins (ESAT6 and CFP10) were used for stimulation of the recall response^20^. Although PPDB and PPDA have overlapping protein antigens, PPDA represented NTM antigens. Exposure to NTM generally does not pose a health threat to animals or humans in immunocompetent individuals. In contrast, responses to PPDB and ESAT6 and CFP10 indicate a recall response to MTBC infection and possible TB infection. Positive responses to PPDB are more common than those against ESAT6 or CFP10, and ESAT6 stimulates IFNγ production at higher levels than CFP10. This finding is consistent with a stronger serological response against ESAT6 in elephants^9^. Using specific antigens, ESAT6 and CFP10 in this assay helps to confirm MTBC infection. Interestingly, the combination of ESAT6 and CFP10 yielded higher IFNγ release than a single protein. This result implies that T cell clones specific to ESAT6 and CFP10 additively enhance IFNγ release and the assay has higher sensitivity when the two antigens are used in combination to stimulate PBMCs.

Although immune responses and disease outcomes of tuberculosis in elephants is not well characterized, it was reported that elephants mounted strong humoral responses to M. tb infection^18^. Asian elephants have higher susceptibility scores to M. tb, compared to African elephants but the underlying immunological differences between species have not been documented^6^. With the ease of testing, the serological diagnosis of elephant TB has been used more often in the field, and a commercial testing kit is available^9,21,22^. In humans, the serological test for TB is not recommended because it can give rise to a false positive due to environmental mycobacterial response. Interestingly, in elephants, specific antibody responses against M. tb antigens (ESAT6/CFP10) declined upon drug treatment^9^. In rhinoceros (*Diceros bicornis*), rising in antibody titer was observed before the death of the animal, while a decrease in antibody titer correlated with treatment^23^. It is not known whether the serological response correlates with the latent or active stage of TB in elephants.

Our study highlighted the discrepancies of serological positivity and IGRA positivity in elephants. In this study, more elephants were found to be IGRA-positive than serologically positive (18 in 32 for IGRA vs. 5 in 32 for serological test). Four out of five animals showed a positive response in both assays. As IGRA is an assay for immunological recall response, the positive result indicates past exposure to MTBC, regardless of whether the animals are in the latent or active stage. In contrast, serological response, at least in elephants, seemed to fluctuate with treatment and, possibly disease stage, and the two tests may complement each other for veterinary care for elephants and for measures to protect humans in close contact with the elephants. As previously stated, IGRA positivity indicates previous exposure to MTBC, but serological response may correlate with disease stage. Therefore, using IGRA for elephants may present a screening test to identify animals that are at risk of developing or transmitting the diseases. On the other hand, serological response may be useful for monitoring response to treatment or disease progression. Interestingly, one animal (sample No. 28) in our study cohort that were tested positive by serological test back in 2014, has recently turned negative by serological test in 2018, while IGRA test yielded consistent positive result. This example highlighted the possibility that HIR may fluctuate, depending on the disease stage, but the response by IGRA is maintained.

This study did not aim to investigate whether IGRA is a better diagnostic tool than the serodiagnosis, or vice versa. But the results highlight the importance of using both assays to complement each other to more correctly identify animals that are previously exposed to M. tb (IGRA) and those that may need veterinarian attention (serological assay).

One disadvantage of IGRA developed in this study is the use of PBMC as the source of responding immune cells. Using PBMC for measuring response to stimulating antigens, helps normalizing the discrepancies in the white blood cell counts among individual elephants, but isolating PBMC is time-consuming and labor intensive when handling large numbers of samples. To improve the user friendliness of the assay, whole blood culture is being explored for future use. Furthermore, shorter incubation and the use of peptides from specific M. tb antigens will be investigated. This similar approach is in use for human IGRA when whole blood is used for stimulation before separating plasma to detect IFNγ.

Using the trunk wash PCR method and serological detection, the study of tuberculosis in captive Asian elephants in Malaysia revealed high seroprevalence in the elephants (20.4%) and their caretakers (24.8%)^24^. The seroprevalence in the Malaysian study and the IGRA positivity in our study are in a similar range, suggesting that Asian elephants in captivity may be predisposed to TB infection, possibly from close contact with humans. The use of IGRA for elephant TB detection presents an alternative to the gold standard method of bacterial culture and provides unequivocal results of the previous exposure to MTBC in elephants. The results obtained with IGRA may be employed to achieve better care of elephants in captive.

## Conclusion

In this study, an IGRA using M. tb antigens was developed to detect MTBC and NTM infection in Asian elephants. Application of this test in more than 60 elephants, the world largest numbers to date, revealed high percentages of MTBC infection. Compared with the serological test, IGRA yielded higher numbers of positive elephants, indicating that captive elephants living close to humans are at higher risk of exposure to MTBC infection. Because IGRA positivity does not imply the disease stage, but the use of IGRA and serological diagnosis as combination tools may provide better way for TB diagnosis and monitoring of diseases in elephants.

## Methods

### Asian elephants

Blood samples (n = 61) were collected from captive Asian elephants in Thailand. Elephants were registered under health checkup program by the Zoological Park Organization and the National Elephant Institute. No exclusion criteria are set for this study. All procedures involving elephants were approved by the Institutional Animal Care and Use Committee (IACUC) of the Zoological Park Organization of Thailand and The National Center for Genetic Engineering and Biotechnology (BT-Animal 07/2560) and were performed according to the guidelines issued by IACUC. The use of mice to generate monoclonal antibodies against recombinant eIFNγ was approved by IACUC of Lab Animal Center of Chulalongkorn University with approved animal use protocol No. 1673038.

### Reagents

Purified protein derivative (PPD) from *M. bovis* (PPDB) and PPD from *M. avium* (PPDA) (both from IDvet, France) were used as representative MTBC and NTM mixed antigens. Recombinant ESAT6 and CFP-10 were prepared using *Escherichia coli* and purified by affinity column chromatography as previously described^25^.

### Cloning of *eIFNγ* and recombinant protein preparation

Recombinant eIFNγ was obtained by construction of an expression plasmid harboring the eIFNγ gene from a female Asian elephant (*Elephas maximas*). Briefly, RNA was prepared from mitogen (ConA)-stimulated PBMCs, and cDNA was synthesized from RNA using the PrimeScript 1st cDNA Synthesis Kit (Takara Bio USA) according to the manufacturer’s protocol. Based on bovine IFNγ sequence (GenBank Accession No. M29867, Sreekumar et al.^26^), primers B-IFNF (5′ CAA CTA CTC CGG CCT AAC TCT CTC 3′) and B-IFNR (5′ AGG ACC ATT ACG TTG ATG CTC TCC G 3′) were used to PCR amplified eIFNγ from cDNA, which was subsequently cloned into pGEM T-easy (Promega, USA) to generate pGEM-BIFN10. Specific primers IFNeleNde5 (5′ TAC ATA TGA CTT TTT TGA AAG AGA TAC 3′) and IFNeleXho3 (5′ TAC TCG AGC GTT GAT GCT CTC CGG C 3′) were used to PCR amplified eIFNγ using pGEM-BIFN10 as template. The PCR product was cloned into the expression vector pET24b (Novagen, USA) to generate pET24-IFN10. *E. coli* BL21(DE3) was transformed with pET24-IFN10. DNA sequences of cloned *eIFN*γ gene from plasmid constructs, pGEM-BIFN10 and pET24-IFN10, were aligned with reference eIFNγ^26^ (GenBank accession: EF203241.1) using Clustal Omega (1.2.0) program (Supplementary Fig. 5). There are seven nucleotides of the cloned eIFNγ different from the reference eIFNγ which resulted in five amino acids changes (Supplementary Fig. 6). The five amino acids of cloned recombinant eIFNγ that differ from the reference gene are E54K, N58D, V104I, S113A, G125V. In addition, the recombinant eIFNγ contained ATG (M) start codon at N-terminus, two additional amino acids (L, E) and six histidine residues at the c-terminus.

Recombinant elephant interferon gamma with C-His6 (eIFNγ-His6) was expressed and purified by Talon metal affinity resin (Takara Bio USA) as described by the manufacturer. Rabbit polyclonal antibody specific for IFNγ was prepared by immunizing rabbits with recombinant eIFNγ, and sera were used for ELISA (Lab Animal Service Unit of Department of Plant Disease, Faculty of Agriculture at Kamphaeng Saen, Kasetsart University, Thailand). Mouse monoclonal antibody against eIFNγ was produced by hybridomas obtained by the somatic cell–cell fusion technique. The antibody was purified by affinity column chromatography. Cells were cultured in RPM1640 medium (GE Healthcare, USA) supplemented with 10% fetal bovine serum (FBS) (GIBCO, Thermo Fisher Scientific, USA), 1% HEPES free acid, 1% sodium pyruvate and 1% penicillin–streptomycin (Thermo Fisher Scientific, USA).

### Peripheral blood mononuclear cells (PBMCs)

Ten milliliters of elephant blood was collected in heparin tubes (BD Vacutainer, Becton Dickinson, USA) and processed within 24 h. PBMCs were isolated by using the density centrifugation method with Ficoll-Paque Premium media solution (GE Healthcare, USA). Cell pellets were resuspended in complete RPMI 1640 media supplemented with 0.05 mM β-mercaptoethanol and used for antigen stimulation immediately after isolation.

### Stimulating PBMCs with mitogen or antigens

PBMCs were seeded at 1 × 10^5^ cells/well in a U-bottom 96-well plate (Thermo Fisher Scientific, USA) in a final volume of 150 µl. PBMCs were stimulated with concanavalin A (ConA) (10 µg/ml) (Sigma Aldrich, USA), PPDB (10 µg/ml), PPDA (10 µg/ml), recombinant ESAT-6 and/or CFP10 (10 µg/ml each) for 72 h in a CO_2_ incubator. Each condition was performed with three replicates. After seventy-two hours of stimulation, culture supernatants were collected to detect eIFNγ by using sandwich ELISA.

### Generating monoclonal antibodies and optimization of sandwich ELISA to detect eIFNγ

To obtain monoclonal antibodies to detect eIFNγ, mice were immunized with recombinant eIFNγ with Freund’s adjuvant and the somatic cell fusion between splenocytes and myeloma cell line P3/NSI/1-Ag4-1 [NS-1] [ATCC TIB-18 (Manassas, VA, USA)] was performed. Hybridoma clones were selected based on indirect ELISA using eIFNγ as coating antigens. The sandwich ELISA was optimized using the combination of rabbit polyclonal antibody as capture antibody and mouse monoclonal antibody as detecting antibody. Firstly, different amounts of mouse monoclonal antibody were tested (Supplementary Fig. 1). Once the optimal dilution was obtained, the addition of goat anti-rabbit IgG was tested. As shown in Supplementary Fig. 2, addition of goat anti-rabbit IgG significantly improved the sensitivity of the assay. Therefore, this formulation was used to detect eIFNγ from elephant PBMC in this study.

### Sandwich ELISA to detect eIFNγ

The 96-well ELISA plates were coated with goat anti-rabbit IgG Fc fragment-specific antibody (Jackson ImmunoResearch, USA). After the blocking step in 10% FBS in PBS, the plate was washed, and diluted rabbit serum specific to eIFNγ (1:5,000) was added to the wells. Next, eIFNγ or samples from culture supernatants were added to the plates. The standard protein eIFNγ was prepared at concentrations of 0.15, 0.31, 0.62, 1.25, 2.5, 5, and 10 ng/ml. The plates were kept overnight at 4 °C. The next day, plates were washed, mouse monoclonal IgG antibody specific to eIFNγ (0.5 µg/ml) was added, and plates were incubated at 37 °C for 1 h. Goat anti-mouse IgG (Sigma-Aldrich, USA) conjugated with peroxidase (1:10,000) was added to each, followed by TMB substrate for detection. The reaction was stopped with 1 M H_2_SO_4_. The absorbance was measured at 450 nm by a microplate reader (Multiskan FC, Thermo Fisher Scientific, USA).

### Determination of the intra-assay coefficient of variation (CV) of the sandwich ELISA

To determine CV of the assay, the sandwich ELISA was performed in triplicates at the same time with the same reagent and samples, while the inter-assay CV was performed with three different assay times, each time the reagents and samples were prepared separately. The eIFNγ was spiked to the media at the final concentration 0.5, 1, 2.5, and 5 ng/ml. Twelve replicates of each concentration were performed by using the sandwich ELISA. The percentage of intra-assay and inter-assay CV (% CV) was calculated using the following formula^27^:

$$\% {\text{ CV}}\, = \,{1}00 \, \times \, ({\text{standard}}\,{\text{deviation}}/{\text{mean}}).$$

### DPP VetTB assays for elephants

The DPP VetTB Assay for Elephant test kit (Chembio Diagnostic Systems Inc, USA) is a single-use immunochromatographic rapid test for the detection of antibodies against MTB and *M. bovis*. The kit was used according to the protocol from the manufacturer. The coated antigens are MPB83 from *M. szulgai* and ESAT6/CFP10^28^. According to the test kit instruction, the results based on the appearance of test lines were interpreted as negative, TB reactive or TB/mycobacteriosis reactive.

## Supplementary information

Supplementary Information.
