# Supplementary material for: Detection of Mycobacterium tuberculosis complex infection in Asian elephants (Elephas maximus) using an interferon gamma release assay in a captive elephant herd
Source: Sci Rep. 2020 Sep 3;10:14551. doi: 10.1038/s41598-020-71099-3 (PMC7471291; doi:10.1038/s41598-020-71099-3)
Supplement: Supplementary file 1 — Supplementary Information. [file 41598_2020_71099_MOESM1_ESM.docx]

**Supplementary Information**

**Detection of *Mycobacterium tuberculosis* Complex Infection in Asian Elephants (*Elephas maximus*) Using an Interferon Gamma Release Assay in a Captive Elephant Herd**

Songkiat Songthammanuphap, Songchan Puthong, Chitsuda Pongma, Anumart Buakeaw, Therdsak Prammananan, Saradee Warit, Wanlaya Tipkantha, Erngsiri Kaewkhunjob, Wandee Yindeeyoungyeon and Tanapat Palaga

**
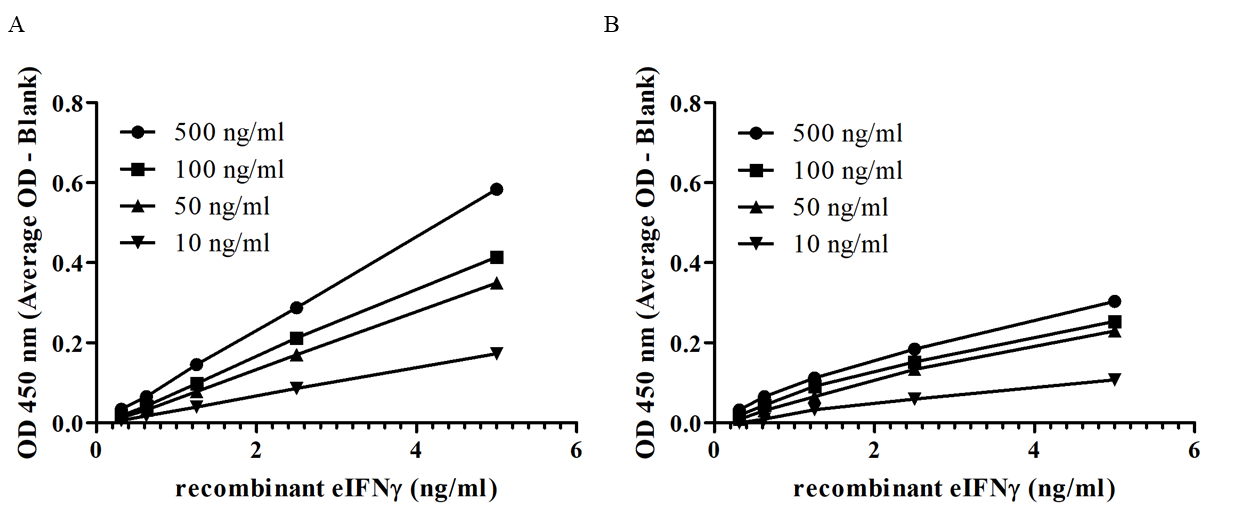
**

**Supplementary Figure 1** Optimization of sandwich ELISA

Two different dilutions of rabbit anti-eIFNγ antibody (A) 1:10000 and (B) 1:25000 were tested in combination with four different concentrations of mouse monoclonal antibody (10, 50, 100, 500 ng/ml) to determine the optimum combinations for use in the sandwich ELISA.


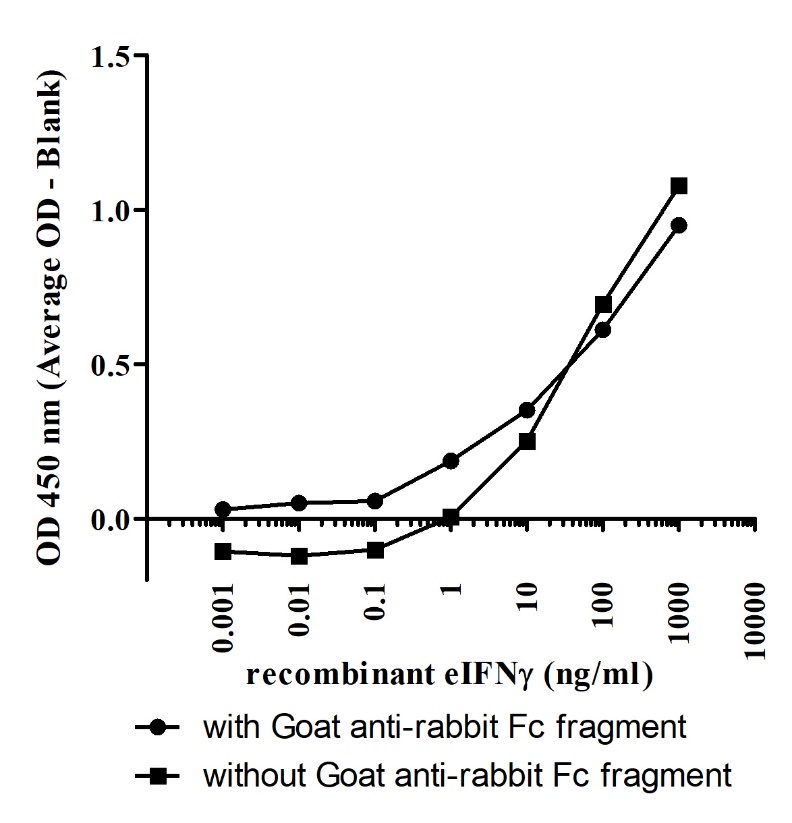


**Supplementary Figure 2** Testing the use of goat anti-rabbit IgG in sandwich ELISA

To test the effect of enhancing sensitivity of the sandwich ELISA using goat anti-rabbit Fc fragment antibody, plates were coated with or without goat anti-rabbit Fc fragment antibody at the dilution 1:1000. Sandwich ELISA was performed using rabbit polyclonal antibody and mouse monoclonal antibody as described above and in Figure 1A.


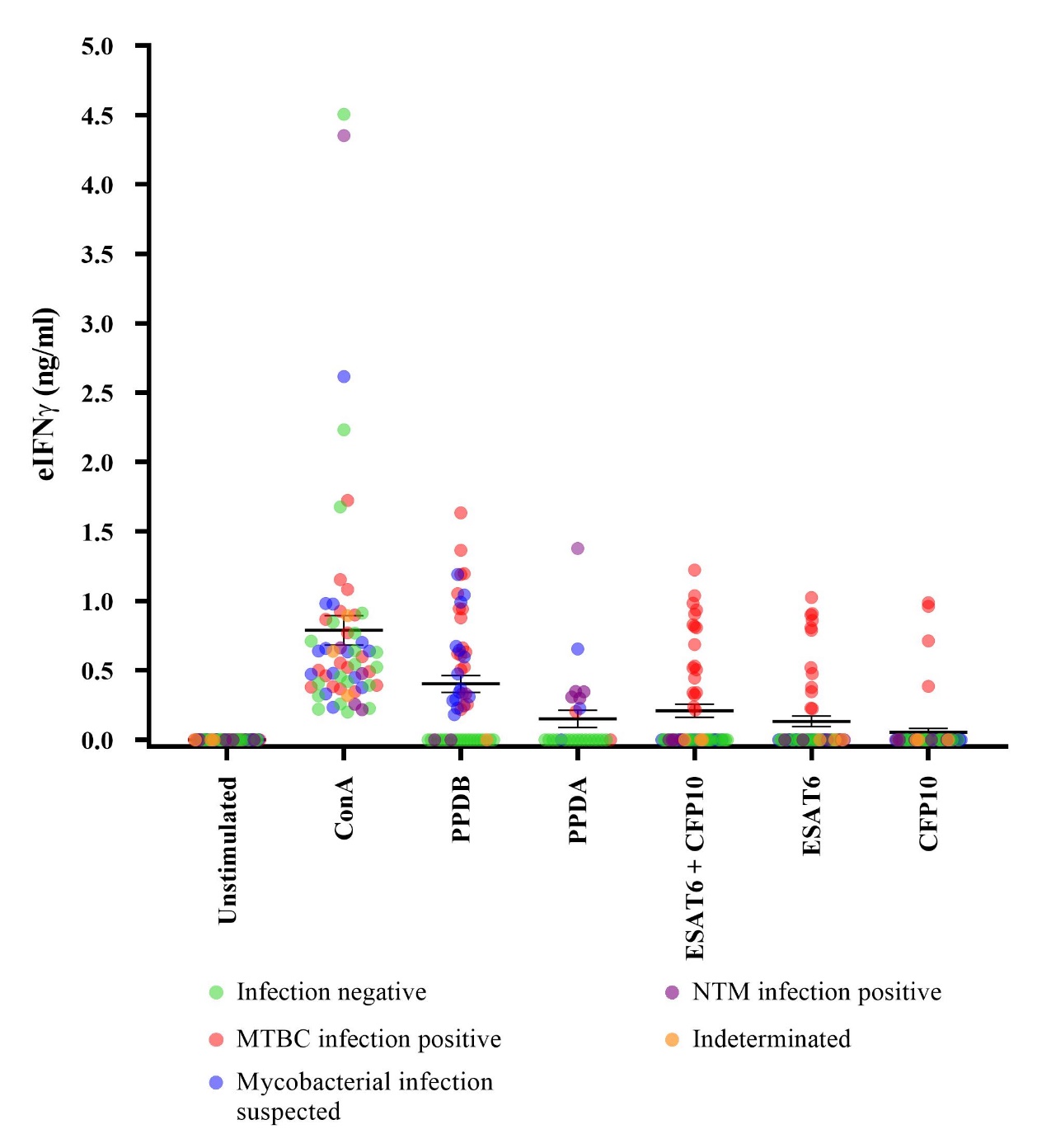


**Supplementary Figure 3** The summary of calculated eIFNγ detected by IGRA

The amounts of eIFNγ detected in the culture supernatant of PMBC culture with different stimulating antigens are summarized. Color coded points represented individual elephant with the infection status based on the criteria depicted in Figure 3.

**Supplementary Figure 4** Comparison of ELISA OD readings when ESAT6, CFP10 or combined antigens were used as stimulating antigens in IGRA

The OD readings from ELISA to detect eIFNγ in culture supernatant of PMBC culture with ESAT6, CFP10 or combined antigens are shown. Lines connecting each dot in different antigens indicated that the samples are from the same individual elephant.

**Supplementary Figure 5** DNA sequence alignment of eIFNγ constructs

Asterisk (*) indicates 7 nucleotides different from reference eIFN**γ** (Sreekumar et al, 2007: GenBank accession: EF203241.1) that resulted in 5 amino acids changes (highlighted in grey); nucleotide sequences added to the primers (to facilitate cloning into an expression vector, pET24b) are underlined; TAA stop codon are double underlined; leader sequences are bolded.

**Supplementary Figure 6** Amino acid sequence alignment of reference and cloned eIFNγ

Predicted amino acid sequence of the cloned eIFNγ gene (pET24-IFN10) was compared to mature sequence of reference eIFNγ (Sreekumar et al, 2007: GenBank accession: EF203241.1). The cloned recombinant eIFNγ on pET24b contains extra 9 amino acids. These 9 amino acids are “M” (start codon), “L”, “E”, and six “H” (histidine) at the c-terminus. The discrepancies of the nucleotide sequences between reference gene and cloned eIFNγ resulted in 5 amino acids (underlined) changes, i.e. E54K, N58D, V104I, S113A and G125V.
